# Improved bleeding scores using Gelfoam^®^ Powder with incremental concentrations of bovine thrombin in a swine liver lesion model

**DOI:** 10.1007/s11239-016-1388-6

**Published:** 2016-06-22

**Authors:** Dennis C. Morse, Elif Silva, Jolee Bartrom, Kelli Young, Eric J. Bass, David Potter, Trevor Bieber

**Affiliations:** 1 Drug Safety Research and Development, Pfizer, 445 Eastern Point Road, Groton, CT 06355 USA; 2 Pfizer, 235 E 42nd Street, New York, NY 10017 USA; 3 NAMSA, 6750 Wales Road, Northwood, OH 43619 USA; 4 NAMSA, 4050 Olson Memorial Highway, Suite 450, Minneapolis, MN 55422 USA; 5 921 Toledo Ave N, Golden Valley, MN 55422 USA

**Keywords:** Gelfoam, Gelatin powder, Thrombin, Hemostasis, Bleeding, Swine

## Abstract

Topical hemostatic agents are used intra-operatively to prevent uncontrolled bleeding. Gelfoam^®^ Powder contains a hemostatic agent prepared from purified pork skin gelatin, the efficacy of which is increased when combined with thrombin. However, the effect of increasing concentrations of thrombin on resultant hemostasis is not known. This study sought to evaluate the ability of various concentrations of thrombin in combination with Gelfoam Powder to control bleeding using a swine liver lesion model. Ten pigs underwent a midline laparotomy. Circular lesions were created in the left medial, right medial, and left lateral lobes; six lesions per lobe. Gelfoam Powder was hydrated with Thrombin–JMI^®^ diluted to 250, 375, and 770 IU/mL. Each concentration was applied to two lesion sites per lobe. Bleeding scores were measured at 3, 6, 9, and 12 min using a 6-point system; comparison of bleeding scores was performed using ANOVA with the post hoc Tukey test. The bleeding scores with thrombin concentrations at 770 IU/mL were significantly lower than at 250 and 375 IU/mL at all four time points. The percentage of biopsies with a clinically acceptable bleeding score rose from 37.9, 46.6, and 71.2 % at 3 min to 55.2, 69.0, and 88.1 % at 12 min in the 250, 375, and 770 IU/mL thrombin groups, respectively. The study showed that the hemostatic response to thrombin was dose-related: using higher concentrations of thrombin with Gelfoam Powder yielded improved hemostasis, as determined by lower bleeding scores.

## Introduction

Maintenance of hemostasis represents a challenge in all surgical procedures. Although a degree of bleeding is commonplace during any surgery, untreated or uncontrolled bleeding can lead to hematoma, infection, repeat surgery, blood transfusion, tissue/organ damage, secondary morbidities, and mortality [1].

Several mechanisms are used to overcome bleeding during surgery, including direct pressure or pressure dressing, electrocautery, sutures, and ligatures. In addition, topical hemostatic agents are highly useful intra-operative interventions; these can be divided into three groups: (1) hemostats, which clot blood, e.g., thrombin; (2) sealants, which prevent leakage, e.g., fibrin sealant; and (3) adhesives, which stick tissues together [2].

Thrombin is a coagulation protein that is available as an active biological hemostatic agent from bovine, human, or recombinant (human) sources. Thrombin activates platelets and converts soluble fibrinogen into insoluble fibrin, thus providing a lattice for platelet aggregation and thrombus formation [3]. Application methods can vary: thrombin can be used in combination with fibrinogen, as a glue to create a seal [4]; delivered as a spray [5]; or applied in combination with a gelatin matrix [6]. Multiple surgical specialists, including cardiac [7], liver [8], spinal [9], and vascular surgeons [10], use thrombin alone or in combination with gelatin products to help control bleeding.

Gelfoam^®^ (Pfizer, New York, NY, USA), is a hemostatic agent prepared from purified pork skin gelatin [11]. It can be used in its dry form, or it can be saturated with saline or thrombin prior to use. It is known that the efficacy of Gelfoam to control bleeding is enhanced when used in combination with thrombin [12]. However, the effect of increasing thrombin concentrations when used with Gelfoam Powder on resultant hemostasis has not previously been investigated.

In the current study, Gelfoam Powder was prepared with various concentrations of a topical bovine thrombin, Thrombin-JMI^®^ (Pfizer, New York, NY, USA). The aim of the study was to evaluate the ability of increasing concentrations of thrombin in combination with Gelfoam to control bleeding in the swine liver lesion model, using a qualitative 6-point scoring system [12].

## Methods

### Study species and definitions

The study was conducted by NAMSA, Northwood, OH, USA, in accordance with FDA Good Laboratory Practice Regulations, 21 CFR 58. All procedures were performed under approval from the NAMSA Animal Care and Use Committee.

**Fig. 1:**
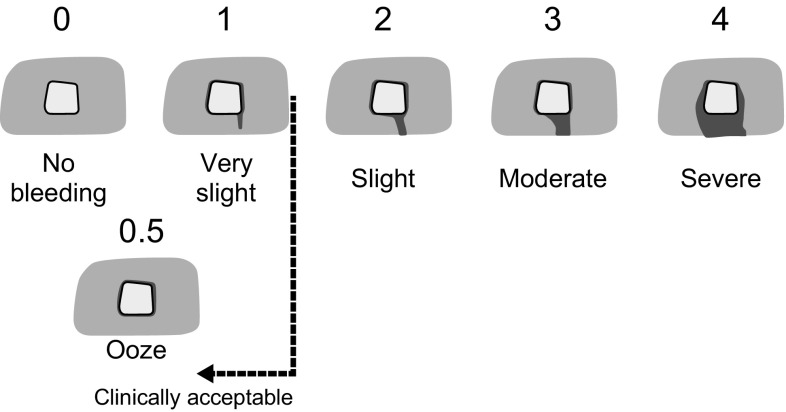
Qualitative, 6-point visual bleeding score system. *Score 0*, no bleeding seen from the liver lesion site; *Score 0.5*, blood observed at the edge of the liver site though not flowing; *Score 1*, blood trickles very slowly from the liver lesion site; *Score 2*, blood flows slowly from the liver lesion site; *Score 3*, blood flow from the liver lesion site is definite; *Score 4*, blood flows freely from the liver lesion site in a steady flow.* Scale* reproduced with kind permission from Springer Science+Business Media [12, Fig. 1]

The study species, the pig, *Sus scrofa domesticus*, was chosen because: (1) the anatomy and physiology of pigs is considered to be more similar to humans than any other laboratory animal; (2) pigs provide a better model for evaluation of bleeding than any other species; and (3) a large animal model is necessary to provide a large enough liver surface area for the number of test sites required. For the purpose of this study, bleeding scores were measured by a visual, qualitative 6-point scale that has previously been used in other studies [1, 12–14] (Fig. 1), whereby the lower the score, the better the indication of hemostasis outcome. Assessments were made by investigators who were blinded to the concentration of product used in each lesion or test article, to maintain objectivity of study findings.

Animals were observed daily for signs of illness, injury, or death and any signs of pain including anorexia, reduced fecal output, vocalization, decreased social interactions, refusal to move, hunched body position, and limping. Additionally general appearance, respiratory system, gastrointestinal system, neurologic system, and musculoskeletal system were also examined. Body weights were recorded on day −1. Any animal that was not considered healthy, based on clinical assessment and body weight, was not selected for use in the study. Data were collected for ten female pigs (Yorkshire cross) aged 16–20 months, each weighing between 70 and 86 kg at surgery. Each animal had six circular lesions made on each of the left medial, right medial, and left lateral lobes of the liver.

The test article was Gelfoam Powder composed of porcine gelatin (Pfizer; Identification SWO 3027 A), prepared with the addition of Thrombin-JMI^®^ (Pfizer; Lot 618952) of three different concentrations. Gelfoam Powder in the amount of 0.550 g was preloaded in a 10 mL syringe and sterilized by gamma irradiation rather than heat sterilization used for the commercially-available Gelfoam Powder [15]. Thrombin-JMI^®^ (20 K IU) was diluted to 1000 IU/mL with a volume of 20 mL of sodium chloride USP solution (SC; Pfizer; Lot 648045, 14-017-T03, 3001016). The Gelfoam Powder was then mixed with three different volumes of the 1000 IU/mL thrombin solution to yield final concentrations of 250 IU/mL (T250), 375 IU/mL (T375), and 770 IU/mL (T770). Only technical personnel preparing the test articles were privy to the thrombin concentrations. Each of the three test article concentrations (T250, T375, or T770) was applied to two liver lesion sites (per lobe). The aim of the study was to evaluate the ability of increasing concentrations of thrombin in combination with Gelfoam to control bleeding in the swine liver lesion model described, using a qualitative 6-point scoring system.

### Coagulation status

Before the liver lesions were created, a baseline blood sample was drawn and activated clotting time (ACT) was measured and recorded for each study animal. Heparin was administered to achieve an ACT of ≥1.5× baseline throughout the procedure. After the last lesion was created, a final ACT measurement was collected.

### Surgical procedure: swine liver lesion model

Food was withheld throughout the day and overnight prior to surgery. General anesthesia was induced with intramuscular injection of tiletamine/zolazepam (Telazol^®^; 4.4 mg/kg) and xylazine (2.2 mg/kg) and maintained using isoflurane. Lactated Ringer’s solution and/or saline was administered intravenously. Animals were ventilated and vital signs (temperature, heart rate, respiration rate, peripheral capillary oxygen saturation, indirect blood pressure) were monitored throughout the procedure.

Each pig underwent a midline laparotomy using standard surgical techniques. The liver was exposed and isolated. Six circular lesions approximately 10 mm diameter × 7 mm depth were created using a biopsy punch instrument to each of the left medial, right medial, and left lateral lobes in each animal. Lesions were spaced 1–2 cm apart, to prevent cross-contamination of sites. Once created, lesion sites took 1–4 s to fill with blood (initial bleeding scores were either 3 or 4). If any site was not bleeding adequately, it was scraped with a blade to increase the flow rate. Where lesion sites were considered unacceptable due to overly severe or inadequate bleeding, a replacement lesion was created. In cases of severe bleeding, gentle digital pressure, ligation with suture, Gelfoam^®^ T770, FLOSEAL, or thrombin was used to control the bleeding, and no further data were collected from these lesions for the study analysis.

The appropriate test article concentration and a saline-moistened gauze sponge were applied to the designated liver lesion sites and held by gentle digital pressure for 25–50 s (with a target of 30 s). The gauze was then removed. Bleeding at each lesion site was scored by the surgeon at 3, 6, 9, and 12 min (time tolerance of ±1.5 min for each time point) using the 6-point bleeding score system (Fig. 1). The surgeon and the surgical team were blinded to the concentration of the test article. The clinically acceptable cut-off score was 1.

After completion of surgical procedures and while under anesthesia, animals were euthanized by intravenous injection of a sodium pentobarbital-based solution, consistent with the recommendations of the American Veterinary Medical Association (AVMA) Panel on Euthanasia [16].

### Statistical methods

The sampling unit for this study was the liver lesion site. Prior to initiation of the study, a sample size analysis was conducted to determine the number of lesions required to provide sufficient power to detect differences in mean bleeding scores between thrombin concentration groups. Bleeding score data from two previous preliminary studies (unpublished data, Pfizer) were used to estimate lesion-to-lesion variability, with standard deviations (SD) ranging from approximately 1 to 1.25. Using the more conservative estimate, SD = 1.25, and assuming a 1-sided 2-sample *t*-test with alpha = 0.05, and 80 % power, a total of 60 lesions per thrombin concentration group would lead to a minimum detectable difference in mean bleeding scores of 0.57.

Statistical analysis of bleeding scores was performed using an ANOVA model created for each time point (3, 6, 9, and 12 min). The bleeding score was the response variable and concentration was a fixed effect with three levels (T250, T375, and T770) and the time 0 bleeding score was a covariate. If the overall model showed a 1-sided statistical difference at the alpha = 0.05 level, Tukey *p*-values (adjusted to account for multiple tests on the same data) were created to determine pair-wise differences between the concentrations. The mean score and SD were calculated for each concentration (T250, T375, and T770) at each time point (3, 6, 9, and 12 min). The percent of scores ≤1, ≤0.5, and 0 were summarized for each concentration.

## Results

Clinical assessments indicated all animals to be healthy and of normal body weight at the initiation of the procedure and all animals survived the procedure. Baseline coagulation parameters (prothrombin time, activated partial thromboplastin time, and fibrinogen level) obtained immediately prior to the onset of the procedure were all within an acceptable range [17] (Table 1). Three pigs gave baseline fibrinogen values that were lower than the typical range (160–390 mg/dL) but were not considered to be abnormal. Baseline coagulation parameters were not collected for one animal. However, coagulation parameters after heparinization and bleeding scores were consistent with those obtained for other animals on the study.

**Table 1:** Baseline coagulation parameters

Animal number	Prothrombin time (s)	Activated partial thromboplastin time (s)	Fibrinogen (mg/dL)
2527	10.7	11.9	217
2529	12.0	11.7	144
2523	11.5	11.7	222
2530	11.0	11.5	180
2528	11.1	11.5	129
2522	11.2	13.7	173
2521	12.0	13.5	131
2524	11.4	12.8	140
2525	11.3	14.0	212
2531^a^			

Reference ranges for normal coagulation activity [17]: Prothrombin time, 10–12 s; Activated partial thromboplastin time, 10–25 s; Fibrinogen, 160–390 mg/dL

^a^Baseline coagulation parameters not collected

A total of ten pigs (mean weight ± SD, 76.1 ± 6.2 kg) were used in the study, and bleeding sites were created by punching circular lesions in their liver while under general anesthesia. Fifty-eight lesion sites were tested with the T250 and T375 concentrations and 59 lesion sites were tested with the T770 concentration, all distributed evenly across all animals and each of the three liver lobes tested. The achieved sample size was determined to be statistically acceptable such that the study could be conducted as planned.

For one animal, only four lesion sites were used for evaluation of T250 and T375 concentrations and only five lesions sites for T770 (rather than the six specified), because the pace of bleeding slowed. However, this had no impact on testing, with the scores elicited from the 13 useable test sites being similar to those elicited from the remainder of the animals in the study. A total of 31 lesion sites were deemed unsuitable for evaluation due to inadequate or uncontrollable bleeding and never received treatment with the test articles.

At one T770 lesion site, the test article slid off before the 30-s application target was reached. The article and gauze were re-applied to the site for 30 s and then removed. The bleeding scores were similar to the remainder of the T770 sites for the animal and were used for evaluation. At one T250 liver lesion site, approximately 0.5 mL of the test article was removed during removal of the gauze sponge. The bleeding scores were slightly higher in comparison to the remainder of the T250 sites for this animal, but the site was used for evaluation. One animal suffered a tear in the most cranial aspect of the left medial liver lobe, which caused the abdominal cavity to fill with an excessive volume of blood. However, this did not impact the scores elicited from the lesion sites on the lobe.

**Fig. 2:**
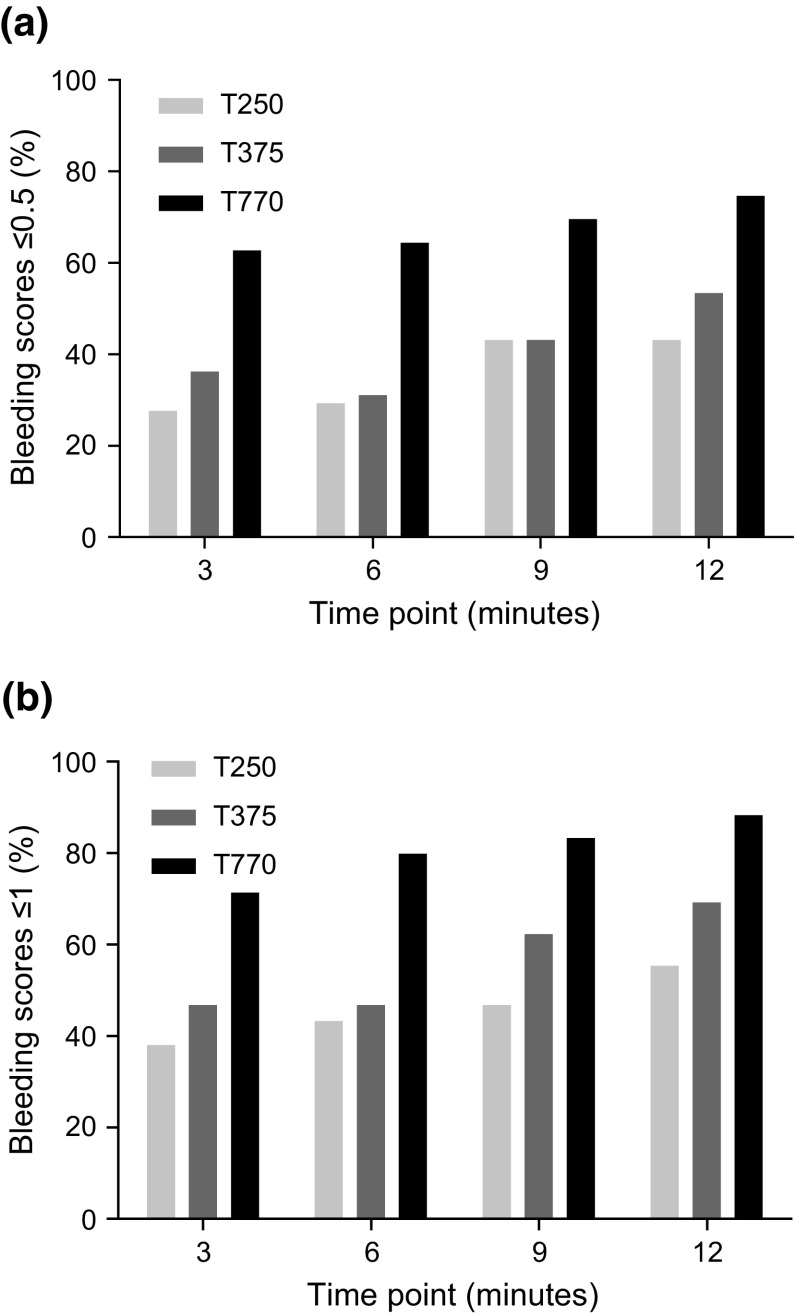
Bleeding scores over time by test article concentration. Bleeding scores measured at 3, 6, 9, and 12 min after removal of the gauze sponge: **a** Bleeding scores ≤0.5; **b** Bleeding scores ≤1

Initial bleeding scores for each test concentration are shown in Table 2. No significant differences were found for initial bleeding score (*p* = 0.7888) or use of additional scraping (*p* = 0.6629) between T250, T375, and T770 concentrations. Bleeding scores measured at 3, 6, 9, and 12 min after removal of the gauze sponge are shown in Table 3 and Fig. 2. After 3 min, 37.9 % of biopsies in the T250 group achieved a clinically acceptable bleeding score (≤1), compared with 46.6 and 71.2 % in the T375 and T770 groups, respectively. By 12 min, the percentage of biopsies achieving a clinically acceptable bleeding score had increased to 55.2, 69.0, and 88.1 % in the T250, T375, and T770 groups, respectively.

**Table 2:** Summary of initial bleeding scores

Parameter	T250, % (n/N)	T375, % (n/N)	T770, % (n/N)
Initial bleeding score			
0	0.0 (0/58)	0.0 (0/58)	0.0 (0/59)
0.5	0.0 (0/58)	0.0 (0/58)	0.0 (0/59)
1	0.0 (0/58)	0.0 (0/58)	0.0 (0/59)
2	0.0 (0/58)	0.0 (0/58)	0.0 (0/59)
3	70.7 (41/58)	72.4 (42/58)	76.3 (45/59)
4	29.3 (17/58)	27.6 (16/58)	23.7 (14/59)
Additional scraping	1.7 (1/58)	0.0 (0/58)	0.0 (0/59)

*N* total number of lesion sites to which each test article concentration was applied; *n* number of lesion sites with each initial bleeding score

**Table 3:** Summary of bleeding scores over time by test article concentration

Scoring interval (min)	T250, % (n/N)	T375, % (n/N)	T770, % (n/N)
Bleeding score of ≤1 (clinically acceptable)			
3	37.9 (22/58)	46.6 (27/58)	71.2 (42/59)
6	43.1 (25/58)	46.6 (27/58)	79.7 (47/59)
9	46.6 (27/58)	62.1 (36/58)	83.1 (49/59)
12	55.2 (32/58)	69.0 (40/58)	88.1 (52/59)
Bleeding score of ≤0.5			
3	27.6 (16/58)	36.2 (21/58)	62.7 (37/59)
6	29.3 (17/58)	31.0 (18/58)	64.4 (38/59)
9	43.1 (25/58)	43.1 (25/58)	69.5 (41/59)
12	43.1 (25/58)	53.4 (31/58)	74.6 (44/59)
Bleeding score of 0			
3	24.1 (14/58)	25.9 (15/58)	57.6 (34/59)
6	15.5 (9/58)	22.4 (13/58)	52.5 (31/59)
9	27.6 (16/58)	29.3 (17/58)	55.9 (33/59)
12	31.0 (18/58)	39.7 (23/58)	67.8 (40/59)

*N* total number of lesion sites to which each test article concentration was applied; *n* number of lesion sites with bleeding scores of ≤1, ≤0.5, or 0 at each time point

**Fig. 3:**
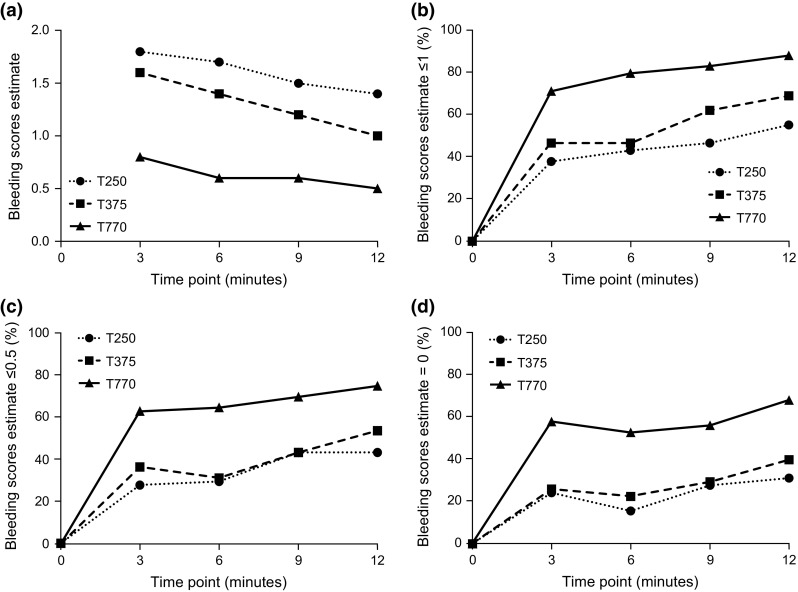
Bleeding scores over time by concentration. **a** Adjusted estimates from the repeated measures linear regression over time by concentration; **b**–**d** Percentage of bleeding scores ≤1, ≤0.5, and =0 by concentration over time where T = 0 is the time at which each lesion was created

The bleeding score was also evaluated using repeated measures linear regression to account for correlation within each animal. The bleeding score estimates and associated standard errors from the regression model are shown in Table 4 and Fig. 3. Using ANOVA with the post hoc Tukey test for multiple comparisons, the estimated bleeding score for the T250 group was significantly higher than the T770 group at all four time points (*p* < 0.0001; Table 4). The estimated bleeding score for the T375 group was also significantly higher than the T770 group at all four time points (*p* < 0.0001 at 3 and 6 min; *p* = 0.0003 at 9 min; *p* = 0.0011 at 12 min). Estimated bleeding scores for the T250 group were not significantly different from bleeding scores for the T375 group at 3, 6, and 9 min (*p* = 0.1764; *p* = 0.0840; and *p* = 0.1507, respectively). However, the estimated bleeding score for the T250 group was significantly higher than the T375 group at 12 min (*p* = 0.0448).

**Table 4:** Comparison of thrombin concentrations using repeated measures linear regression

Time point (min)	Bleeding score estimate (SE)			*p* value*		
	T250	T375	T770	T250 versus T375	T250 versus T770	T375 versus T770
3	1.8 (0.2)	1.6 (0.2)	0.8 (0.2)	0.1764	<0.0001**	<0.0001**
6	1.7 (0.2)	1.4 (0.2)	0.6 (0.1)	0.0840	<0.0001**	<0.0001**
9	1.5 (0.2)	1.2 (0.2)	0.6 (0.2)	0.1507	<0.0001**	0.0003**
12	1.4 (0.2)	1.0 (0.2)	0.5 (0.2)	0.0448**	<0.0001**	0.0011**

* Tukey *p* values adjusted for multiple comparisons

** Significant at 0.05 level

## Discussion

The aim of this study was to investigate the effect of increasing concentrations of bovine thrombin in combination with Gelfoam Powder on resultant hemostasis using a swine liver lesion model. The study showed that addition of higher concentrations of thrombin to Gelfoam Powder yielded improved hemostasis outcomes, as determined by lower bleeding scores. The hemostatic response to thrombin was dose-related: the 770 IU/mL thrombin concentration was associated with statistically lower bleeding scores at each time point compared with either the 375 or 250 IU/mL concentrations.

This is the first in vivo study to directly demonstrate the positive effect of increasing concentrations of thrombin on the hemostatic capability of an absorbable gelatin formulation, in this case the Gelfoam Powder. The standardized protocol used a biopsy punch instrument to create circular puncture lesions of consistent diameter but with greater depth compared with those in other published studies [1, 12]. Furthermore, the study utilized an ordinal, qualitative, 6-point bleeding score system [12] that permitted a more rigorous statistical analysis compared with those studies utilizing a ‘bleeding’ versus ‘no bleeding’ assessment as the only outcome measure. As an additional measure, objectivity of current study data was also maintained by utilizing a “blinded” assessment protocol, so that the bleeding scores were rated by investigators who were blinded to the individual product concentrations of test articles as applied to various lesions.

The results presented here support a previous study in which a Gelfoam sponge used in combination with human thrombin was shown to be more efficacious in controlling bleeding over multiple time points when compared with Gelfoam used in combination with saline control [12]. Notably, the study used a lower single concentration of thrombin (125 IU/mL); and also, the lesions were 3 mm in depth, compared with 7 mm in the current study. The findings of this and the present study are complementary, demonstrating that the addition of thrombin increases the effectiveness of different preparations of Gelfoam.

The swine liver lesion model is useful for testing hemostatic interventions for moderate bleeding during surgical procedures [1, 12]. In the current study, initial bleeding scores were ≥3 prior to the application of test article for all Thrombin–JMI concentrations tested, which allowed the investigators to be able to note macroscopically measurable differences when present between the pre-application and post-application bleeding scores for the administration sites. Further investigations to confirm how the results of this study would apply to hemostatic outcomes for lower degrees of bleeding would be warranted.

The main limitation of the current study was that it tested a single tissue and lesion type. Future studies could therefore also seek to confirm the present findings using other tissues, including those from humans, and with greater diversity of lesions, akin to those encountered during human surgical procedures, or using any other bleeding sites for which the hemostatic products would be applicable for use in medical practice. An advantage of the current study is that it used a larger sample size (n = 10 pigs) than other similar studies and, consequently, had greater statistical power than historical models [1, 12]. Additionally, the choice of standardized model in the current study was clinically relevant since gelatin and thrombin products are frequently used, both individually as well as in combination, in liver surgery.

Another potential limitation of the current study might be the absence of a Gelfoam-only control study arm. Historically, on the other hand, Adams et al. [12] demonstrated the superiority of Gelfoam sponge with 125 IU/mL human thrombin compared with Gelfoam sponge alone in a swine liver lesion model. Future studies that explore comparative hemostatic outcomes of various combination hemostatic treatments might similarly consider inclusion of a Gelfoam-only arm as necessary to further explore specifics of incremental value.

The current study examined the clinically recommended concentration of 1000 IU/mL of Thrombin JMI in the 770 IU/mL arm of the study, as the addition of the Gelfoam powder to the 1000 IU/mL of this thrombin yielded a final concentration of 770 IU/mL of mixed product in test article. Lower (100 IU/mL) and higher ranges of the clinically recommended concentrations of Thrombin–JMI (up to 2000 IU/mL), and even more interestingly concentrations above 2000 IU/mL, might also be explored in future studies to confirm whether the resultant data would follow the dose-proportional hemostatic response trend obtained in the current study. Exploring the concentration ranges for all thrombins, used either alone or in combination with other hemostatic agents, on various tissues and surgical situations might be of particular interest for any future pre-clinical and clinical studies.

The current study employed bovine thrombin (Thrombin–JMI 20,000 IU vials, Pfizer) in combination with Gelfoam Powder (Pfizer). Bovine thrombin is still used routinely, while over the past decades, human and recombinant (human) thrombin alternatives have also been developed [3, 5]. Despite the current availability of various thrombin formulations of different origins for human use, effects of varying concentrations of thrombin on hemostatic outcomes in different tissues have rarely been investigated, and even less in combination with absorbable gelatin powder. A comparative study investigating the differences in efficacy by addition of varying concentrations of bovine, human, or recombinant (human) thrombin to Gelfoam would therefore be warranted. Indeed as tested in a porcine model, the results of a comparative study between bovine- and porcine-derived gelatin in combination with human thrombin demonstrated that the different sources of gelatin also accounted for variations in hemostatic efficacy, reportedly due to differences in ultrastructure [1].

In conclusion, the current study showed that higher concentrations of thrombin used in combination with absorbable gelatin powder yields better hemostatic results in the swine model as demonstrated by the bleeding scores. As this is the first study ever conducted to look at differential hemostatic findings between various thrombin concentrations, future studies to further investigate the applications of these findings in other tissues and also comparisons between thrombins and absorbable gelatins of different origins are therefore warranted to further refine surgical approaches to hemostasis.
